# Clinical Significance of Serum Interleukin-31 and Interleukin-33 Levels in Patients of Endometrial Cancer: A Case Control Study

**DOI:** 10.1155/2016/9262919

**Published:** 2016-05-31

**Authors:** Xi Zeng, Zhu Zhang, Qian-Qian Gao, Yan-Yun Wang, Xiu-Zhang Yu, Bin Zhou, Ming-Rong Xi

**Affiliations:** ^1^Department of Gynecology and Obstetrics, The West China Second University Hospital, Sichuan University, Key Laboratory of Birth Defects and Related Diseases of Women and Children (Sichuan University), Ministry of Education, Chengdu 610041, China; ^2^Department of Molecular Translational Medicine, The West China Institute of Women and Children's Health, West China Second University Hospital, Sichuan University, Chengdu, China

## Abstract

*Aims*. Previous evidence has proved that interleukin-31 (IL-31) and interleukin-33 (IL-33) can be potential markers in some cancers' formulation. We aimed to determine the potential role of IL-31 and IL-33 in prognosis of endometrial cancer patients.* Methods*. Serum samples were collected from 160 patients with endometrial cancer and 160 healthy controls. The ELISA kits (Raybio® Systems) specific for human IL-31 and human IL-33 were used. Serum levels of tumor markers (CEA, CA-125, and CA19-9) were measured by chemiluminescence immunoassay. A two-side* P* value < 0.05 was indicated to be significant.* Results*. Serum levels of IL-31 and IL-33 in patients were significantly elevated compared to those of healthy controls. The interleukin levels were also related to clinical characteristics, including tumor stages, depth of invasion, and existence of node metastases and distant metastases. The sensitivity and specificity of IL-31 and IL-33 were higher than the counterparts of tumor markers, both separately and in combination of IL-31, IL-33, and the clinical markers.* Conclusions*. This report is the first one mentioning the possible association between serum IL-31 and IL-33 and endometrial cancer. With their sensitivity and specificity, the interleukins may be useful biomarkers for endometrial cancer's prognosis.

## 1. Introduction

Endometrial cancer (EC) is the second most common female cancer with incidence varying among regions. More than 287,100 new EC cases are diagnosed each year, which are more common in developed countries with the risk of endometrial cancer being 1.6% and oppositely less common in developing countries with the lowest rates of 0.6% [1].

EC occurs most frequently during perimenopause and menopause, between the ages of 50 and 65 worldwide [2]. The worldwide median age at diagnosis of endometrial cancer is 63 years of age [3]. Based on a recent report, EC commonly occurs earlier in Chinese women than those in western countries [4].

The mechanism for the formulation of EC is still not well clear. Endometrial inflammation is considered as an important risk factor for EC [5]. Pathologic angiogenesis has been implicated on the development of chronic inflammatory diseases and several cancers. Some cytokines involved mediating between inflammation and angiogenesis, including interleukins [6, 7]. Previous researches have already revealed neoangiogenesis closely associated with the growth and the development of metastasis of various tumors, including EC [8, 9].

The diagnosis of EC is generally according to the clinical manifestation like postmenopausal bleeding or the serum levels of some tumor markers, while about 15% of ECs occur in women without vaginal bleeding [10]. And the role of tumor markers in endometrial cancer is still in research. Previously, literatures reported the role of different serum markers in endometrial cancer such as carcinoembryonic antigen (CEA), carbohydrate antigen-125 (CA-125), and carbohydrate antigen 19-9 (CA19-9), resulting in elevation in only 20% to 30% of patients [11]. Thus, some more efforts should be exerted on finding a new marker with better sensitivity and specificity in the early diagnosis of endometrial adenocarcinoma.

According to the relationship between the inflammation, angiogenesis, and EC, we supposed that some cytokines perhaps could be used as new markers.

Interleukin-31 (IL-31) belongs to the IL-6 family cytokines and is mainly secreted by activated CD4^+^ T helper (Th) cells [12]. IL-31 acts through the heterodimeric receptors of IL-31 (IL-31R) and oncostatin receptor (OSMR) expressed on IL-31 activated monocytes and expressed on epithelial cells [13]. IL-31 can significantly upgrade the gene and protein expression of vascular endothelial growth factor (VEGF) and epidermal growth factor (EGF) [14].

Interleukin-33 (IL-33) is a new member of the IL-1 family of cytokines [15]. IL-33, passing signal via ST2 receptor, is considered closely associated with IL-18, one of the most novel members in the family [16]. Previous researches illustrated that the elevated serum IL-18 played an important role in a variety of cancers [17–19], and a similar function of IL-33 is still under debate. Some following researches detected elevated serum IL-33 in various tissues of lung, stomach, spinal cord, skin, and brain, also in cells like epithelial cells lining bronchus and smooth muscle cells [20]. Küchler et al. implied that the expression of IL-33 is regulated by vascular endothelial cells in vivo [21]. Choi et al. [22] reported that IL-33 promotes angiogenesis.

Thus, researches labeled IL-31 and IL-33 with antiproliferative and angiogenic factors. More following evidences have proved that IL-31 and IL-33 play an important role in some cancers' formulation, like lung cancer, gastric cancer, and squamous cancer [23–25]. However, there is no research on the relationship of IL-31 and IL-33 with EC.

In this study, the serum levels of IL-31 and IL-33 in EC patients were studied and assessed the interrelationship with clinical significance and some tumor markers. We also measured levels of these cytokines in healthy persons. This study provides new insight into the mechanism of function of new angiogenic cytokines in EC. Based on English literature reviews, this is the first research reported on the association between IL-31 and IL-33 and EC.

## 2. Materials and Methods

### 2.1. Patients and Blood Samples

After acquiring the consent from ethics committee of the West China Second University Hospital, Sichuan University, China (number 2013-036), we selected 160 patients of EC aged between 18 and 80 years who were admitted to our hospital from Jan. 2014 to Jan. 2015. Those patients had been diagnosed by curettage of uterus and were double checked by two pathologic doctors. The stage of the tumor was classified based on the International Federation of Gynecology and Obstetrics (FIGO) criteria for adenocarcinoma of the endometrium (FIGO, 2009) [26]. There was no radiation or chemotherapy before.

Another 160 sex and age matched females, seeking for physical examination at our hospital, were recruited as healthy controls, who had no history of adenocarcinomas or gynecological diseases. The results of gynecologic examinations and transvaginal sonographies in the controls were normal.

All the subjects consented to join this test which was done in agreement with the Declaration of Helsinki. With obtaining written informed consent, a 7 mL blood sample was collected from the antecubital vein and stored at −80°C until testing. Throughout the research, the patients' anonymity was preserved.

### 2.2. Detection of Interleukins and Tumor Markers (CEA, CA-125, and CA19-9)

The ELISA kits (Raybio Systems) specific for human IL-31 and human IL-33 were used to detect serum IL-31 and IL-33 levels, respectively. The sensitivity limits of the ELISA kits were separately from 4.92 pg/mL to 1200 pg/mL (IL-31) and from 2.05 pg/mL to 500 pg/mL (IL-33). The intra-assay and interassay reproducibilities of the ELISA kits were 9% and 11% (IL-31) and 8% and 11% (IL-33). The levels of tumor markers (CEA, CA-125, and CA19-9) were measured by chemiluminescence immunoassay. Our clinical laboratory has been certificated with ISO15189. The reagents manufactured by SIEMENS (Massachusetts, USA) were used in the test of the tumor markers. The sensitivity limit of the CEA reagent was from 0.5 ng/mL to 100 ng/mL (interassay reproducibility 3.3%, intra-assay reproducibility 3.8%). The CA19-9 reagent's sensitivity limit was from 1.2 U/mL to 700 U/mL (interassay reproducibility 8%, intra-assay reproducibility 4%). The sensitivity limit of the CA-125 reagent was from 2 U/mL to 600 U/mL (interassay reproducibility 4%, intra-assay reproducibility 3%). The normal standards were set as follows: CEA < 2.5 ng/mL, CA-125 < 35 U/mL, and CA19-9 < 30 U/mL. All tests were performed based on the instruction.

### 2.3. Statistical Analysis

The SPSS software (Version 22.0, IBM Company, USA) was used for data analysis. Data is shown as mean ± standard deviation (range). The categorical variables between the patients and controls were performed with chi-squared tests. After a normality test of the original data, the correlation between the clinical factors and the levels of IL-31 and IL-33 was analyzed by independent-samples *t*-test. The correlation between the tumor markers and the interleukin levels was analyzed with Student's *t*-test. A cut-off value was determined by analysis of receiver operating characteristic (ROC). Logistic analysis was adopted in the multitarget diagnostic value evaluation. A two-side *P* value < 0.05 was indicated to be significant. The figures were made by the GraphPad Prism 5 software package (GraphPad Software Inc., USA).

## 3. Results

### 3.1. Serum IL-31 and IL-33 in Patients and Controls

The mean age of diagnosis in EC was 54.13 years (range from 37 to 74 years). To be specific, there were 44 patients with tumor stage I; 44 patients had stage II disease; 32 patients were with stage III; and 40 patients were with stage IV. The healthy controls were with a median age of 54.03 years (range from 38 to 74 years) (Table 1).

**Figure 1:**
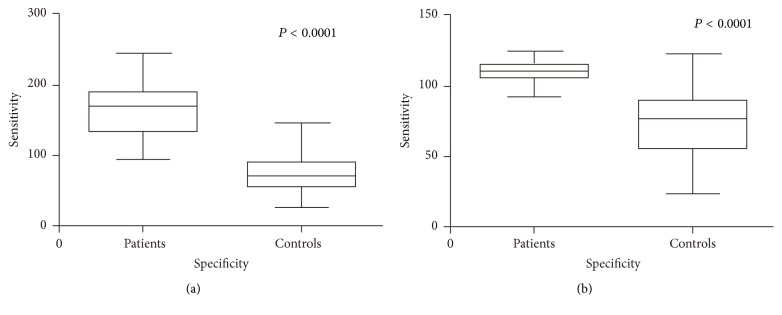
(a) Comparison of the serum levels of IL-31 between the patients and controls. (b) Comparison of the serum levels of IL-33 between the patients and controls.

**Figure 2:**
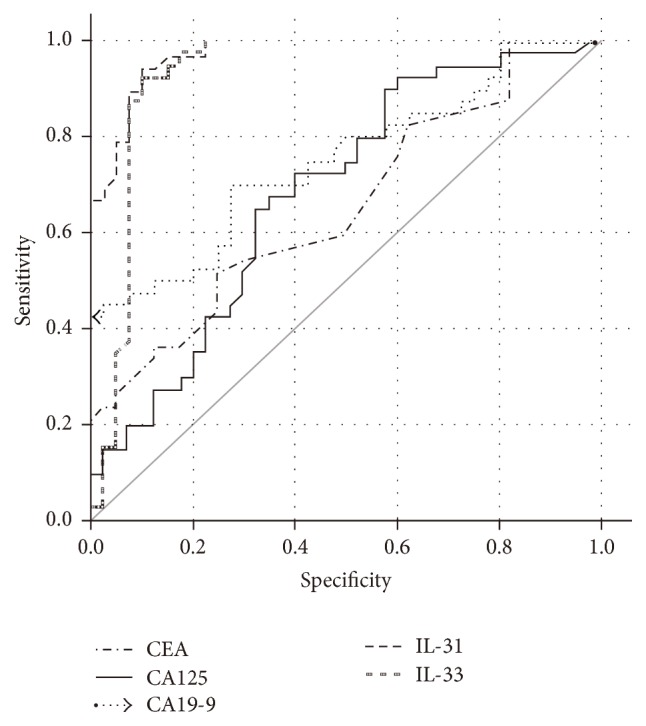
Comparison of the ROC curves among the serum levels of CEA, CA-125, CA19-9, IL-31, and IL-33.

There was no age difference found between patients and controls (*P* = 0.681). A seven-point standard curve was drawn for estimating the concentration of serum IL-31 (*R* ^2^ = 0.998) and IL-33 (*R* ^2^ = 0.999). The results of serum level of IL-31 and IL-33 in EC were shown in Table 2. Both of the serum levels of IL-31 and IL-33 in the patients were dramatically higher than the counterparts of the controls (for IL-31, *P* < 0.0001; for IL-33, *P* < 0.0001; Figure 1). The cut-off value of serum IL-31 was about 113.1 pg/mL (sensitivity: 92.68%, specificity: 94.87%) and the area under the ROC curve (AUC) of IL-31 was 0.973; *P* < 0.0001; 95% CI: 0.945–0.998. The best cut-off value of serum IL-33 was about 98.42 pg/mL (sensitivity: 88.64%, specificity: 97.22%); the AUC was 0.929; *P* < 0.0001; 95% CI: 0.86–0.998 (Figure 2).

### 3.2. Relationship between Serum IL-31/IL-33 and Clinicopathologic Factors

The clinical characteristics include the age, BMI, tumor stages, depth of invasion, lymphaden metastasis, and distant metastasis. The associative results between serum levels of IL-31 and IL-33 and clinical factors in patients with EC were listed in Table 3. It revealed that serum IL-31 was related to tumor stages (*P* = 0.024) and serum IL-33 was close to the disease process: tumor stages (*P* = 0.035), depth of invasion (*P* = 0.008), and existence of node metastases (*P* = 0.029) and distant metastases (*P* = 0.036). However, to our knowledge, there are no publications on the clinical characteristics of IL-31/IL-33 in EC.

### 3.3. Serum Levels of Tumor Markers

Furthermore, we analyzed the ROC of the tumor markers in the EC patients. The mean concentration of serum CEA level was 5.22 ± 14.33 ng/mL (sensitivity: 80%, specificity: 45.71%), and the AUC was 0.644; *P* = 0.027; 95% CI: 0.524–0.764. The mean concentration of serum CA-125 level was 25.80 ± 17.78 U/mL (sensitivity: 72.72%, specificity: 46.38%), and the AUC was 0.684; *P* = 0.005; 95% CI: 0.567–0.801. The mean concentration of serum CA19-9 level was 60.69 ± 63.54 U/mL (sensitivity: 81.33%, specificity: 47.86%), and the AUC was 0.751; *P* < 0.001; 95% CI: 0.645–0.857 (Figure 2). Based on our data, there seemed to be no relationship between the serum levels of the interleukins and the tumor markers (data not shown). Another logistic analysis of multitarget diagnostic value evaluation verified that the sensitivity and specificity combination of IL-31 and IL-33 were also higher than the counterparts of tumor markers (Table 4).

## 4. Discussion

Tumor growth and metastases are regulated by several pathological processes and mediated by inhibitors and stimulators. The close association between inflammation and cancer has been well studied in that the inflammation orchestrates the tumor microenvironment [27, 28]. Previously, researches have established that T cell-mediated inflammation plays a major role in the development of most cancers.

IL-33 proved signaling via the receptor-related protein of ST2L, which is widely expressed on Th cells, and mast cells and the receptor can be higher in cancer patients than in healthy controls [10, 20, 29]. IL-33 can be produced by necrotic or inflamed tissues and used as an alarmin for damaging inflammation [30]. Sun et al. reported that IL-33 levels in the serum of gastric cancer patients were significantly elevated in comparison with that of healthy volunteers, and higher serum levels of IL-33 in gastric cancer patients were found to correlate with several poor prognostic factors like depth of invasion, distant metastasis, and advanced stage and serum immunoglobulin levels can be elevated by either stimulation of cells with IL-33 or binding of IL-33 to the ST2L in the process of some cancers [25]. To the tumor specific immune response, it can also be turned down or even blinded by some Th2 type cytokines [30]. Therefore, IL-33 can join in Th2 immunity and stimulate the ST2L receptor on various inflammatory cells, playing an important role in the release of proinflammatory factors [31]. Following those factors, vascular changes can be induced, including upgrading the permeability of the microvasculature and vasodilatation and recruiting inflammatory cells [32].

Furthermore, IL-33 is considered closely associated with IL-18 [16], which has been demonstrated novelly to be upregulated in cancer patients [33]; thus IL-33 may also highlight a close association with tumor. Previously, researches had confirmed it in gastric cancer patients [25].

In the cancer group, we found that the serum level of IL-33 was higher than in controls (*P* < 0.0001). We also found that the clinical characteristic status correlates with the IL-33 values. With a more malignant factor, the serum IL-33 got a higher level and was associated with the tumor stages (*P* = 0.035), depth of invasion (*P* = 0.008), distant metastasis (*P* = 0.014), and lymph node metastasis (*P* = 0.029). The results confirmed our former hypothesis that serum IL-33 developed with the process of EC. In particular, IL-33 cut-off of 98.42 pg/mL yields the best sensitivity of 88.64% and specificity of 97.22%. In our study, the serum IL-33 is commonly elevated accompanied with some serious clinical factors, like higher tumor stages, depth of invasion, and existence of lymph node metastasis and distant metastasis, which may be further suggested to be related to the progress of the adenocarcinoma.

In tumor patients, IL-31 often produced by damaged and inflamed tissues, especially human mast cell, can also join the T helper type 2 (Th2) responses [34, 35]. In EC patients of our study, although the serum level of IL-31 was higher than the one in controls, the internal relations with the clinical characteristics were less clear than the counterparts of IL-33. We hypothesized that the IL-31 may be secreted by mast cells after IL-33 stimulation. Comparing with the tumor markers of CEA, CA-125, and CA19-9, the serum IL-31 is still equipped with a better sensitivity (92.68%) and specificity (94.87%), using a cut-off value at 113.1 pg/mL.

In literature, preoperational tumor markers' levels of CA-125, CEA, and CA19-9 were related to the stage of the disease, myometrial invasion depth, peritoneal cytology, and lymph node metastasis [36–38]. However, the role of those markers in diagnosis of EC is unsatisfied.

CA-125 is a glycoprotein tumor antigen related to epithelial ovarian cancer, which is particularly used for monitoring disease activity for follow-up [39]. Several investigators have presented a sensitivity varying from 24% to 90% in diagnosis of EC [38]. However, the up-expression of serum CA-125 and CA19-9 was detected in only 10% of patients of EC with stages I and II [40]. The following studies illustrated that among the EC patients only 15% of stage I, 33% of stage II, and 62% of stage III have elevated CA-125 levels [41]. CA19-9, a marker related to gastrointestinal cancers, has a sensitivity varying from 11% to 51.5% and a poor specificity in EC [38]. CEA is a marker discovered in patients with colon adenocarcinoma, which is increased in 35% of patients with EC, relating to the size of the uterus and stage of disease [42], and there seems no correlation between lymphoplasmacytic infiltrations of tumor cells and the serum level of CEA in EC [43].

In our research, the sensitivity of CEA was about 80% and specificity was 45.71%, the sensitivity and specificity of CA-125 were about 72.72% and 46.38%, and the counterparts of CA19-9 were 81.33% and 47.86%, which were confirmed with the literature and lower than the IL-33 and IL-31.

In addition, we were unable to illustrate the correlation between IL-31/IL-33 and endometritis. Those patients with endometritis or pelvic inflammation are commonly admitted to the outpatients department, diagnosed by physical examination, and cured by nonoperation methods; thus we failed to acquire enough cases of peripheral blood and tissues of endometritis to set another control group and there seemed to be no similar studies published before. The further implicit function—promotor of carcinogenesis or reflection of systemic inflammation—remains to be clarified.

Because most patients received operation less than 18 months, the follow-up studies are still undertaken, and the correlation between IL-31/IL-33 and the overall survival time of the endometrial cancer patients is still under research.

## 5. Conclusion

To our knowledge, this is the first clinical research describing a possible relationship between IL-31 and IL-33 and EC. Comparing sensitivity and specificity of some tumor markers, we suppose that elevated serum IL-33 and IL-31 levels, especially IL-33, may relate to the process of EC and could be useful biomarkers for the diagnosis of that disease. However, large prospective clinical studies involving ICH with endometritis tissues need to be further researched to check the role of the cytokines as new potential tumor markers in EC.

## Figures and Tables

**Table 1:** Description characters of the patients and controls. Data is shown as mean ± standard deviation (SD).

Characters	Number of patients (%)	Number of controls (%)	*P* value
Sample size	160	160	
Mean age ± SD (range) (years)	54.13 ± 7.78 (37–74)	54.03 ± 7.94 (38–74)	0.826
BMI mean ± SD (kg/m^2^)	24.71 ± 2.76	24.88 ± 2.76	0.948
Family history of cancer			0.136
Yes	24 (15%)	8 (5%)	
No	136 (85%)	152 (95%)	
Menopausal status			0.651
Premenopausal	88 (55%)	96 (60%)	
Postmenopausal	72 (45%)	64 (40%)	
History of pregnancy			0.556
Yes	152 (95%)	148 (92.5%)	
No	8 (5%)	12 (7.5%)	
Uterine bleeding			
Yes	120 (75%)		
No	40 (25%)		
FIGO stage			
I	44 (27.5%)		
II	44 (27.5%)		
III	32 (20%)		
IV	40 (25%)		
Histology			
Adenocarcinoma	160 (100%)		
Nonadenocarcinoma	0 (0%)		

**Table 2:** Description of the serum levels of IL-31 and IL-33 in patients and controls (pg/mL). Data is shown as mean ± standard deviation (range).

Interleukins	Before operation	After operation	*P*_1_ value	Controls	*P*_2_ value
IL-31	165.80 ± 39.03	128.91 ± 29.48	<0.001	77.24 ± 25.85	0.005
	(94.43–240.65)	(53.54–187.54)		(32.50–142.82)	
IL-33	108.85 ± 7.37	80.93 ± 9.47	0.023	74.29 ± 22.92	<0.001
	(90.28–123.10)	(58.22–107.63)		(31.96–120.11)	

*P*_1_ value means the results comparing the interleukin levels in patients with their samples acquiring before and after the operation.

*P*_2_ value means the results comparing the interleukin levels with the patients' samples acquiring before the operation and those of the controls.

**Table 3:** Correlations of serum IL-31 and IL-33 levels with clinical factors in patients with EC (pg/mL). Data is shown as mean ± standard deviation (SD).

Characters	Number of patients (%)	Serum IL-31	*P*_1_ value^*∗*^	Serum IL-33	*P*_2_ value^*∗∗*^
Age (years)					
<60	108 (67.5%)	170.87 ± 33.05	0.335	110.36 ± 7.69	0.062
≥60	52 (32.5%)	181.37 ± 29.11		105.73 ± 7.70	
BMI					
18.5 ≤ BMI ≤ 24.99	72 (45%)	162.76 ± 37.98	0.859	108.11 ± 8.36	0.818
25 ≤ BMI ≤ 28	68 (42.5%)	169.85 ± 38.89		109.71 ± 6.77	
BMI > 28	20 (12.5%)	163 ± 50.57		108.6 ± 6.6	
Tumor stages					
S1 and S2	88 (55%)	164.18 ± 22.09	0.024	106.66 ± 6.27	0.035
S3 and S4	72 (45%)	186.63 ± 37.80		111.54 ± 7.88	
Depth of invasion					
T1 and T2	108 (67.5%)	169.04 ± 29.38	0.182	106.53 ± 6.27	0.008
T3 and T4	52 (32.5%)	183.01 ± 34.84		112.72 ± 7.62	
Lymph node metastasis					
Absent	124 (77.5%)	169.86 ± 27.08	0.104	107.50 ± 4.45	0.029
Present	36 (22.5%)	189.52 ± 37.85		113.52 ± 9.27	
Distant metastasis					
M0	120 (75%)	169.50 ± 29.51	0.100	107.24 ± 6.20	0.014
M1	40 (25%)	188.63 ± 35.80		113.71 ± 8.76	

^*∗*^ *P*_1_ value means the results of IL-31 with the clinical factors.

^*∗∗*^ *P*_2_ value means the results of IL-33 with the clinical factors.

**Table 4:** The value evaluation of multidiagnostic indicators.

Indicators	AUC	Sensitivity (%)	Specificity (%)
IL-31 + IL-33	0.931	93.86%	97.31%
CEA + CA125	0.701	81.32%	51.17%
CEA + CA19-9	0.751	82.01%	60.72%
CA125 + CA19-9	0.807	85.44%	67.99%
CEA + CA125 + CA19-9	0.807	87.56%	76.32%
